# Blocking TCR restimulation induced necroptosis in adoptively transferred T cells improves tumor control

**DOI:** 10.18632/oncotarget.12674

**Published:** 2016-10-14

**Authors:** Pravin Kesarwani, Paramita Chakraborty, Radhika Gudi, Shilpak Chatterjee, Gina Scurti, Kyle Toth, Patt Simms, Mahvash Husain, Kent Armeson, Shahid Husain, Elizabeth Garrett-Mayer, Chethamarakshan Vasu, Michael I. Nishimura, Shikhar Mehrotra

**Affiliations:** ^1^ Department of Surgery, Medical University of South Carolina, Charleston, SC, USA; ^2^ Department of Microbiology & Immunology, Medical University of South Carolina, Charleston, SC, USA; ^3^ Department of Surgery, Loyola University, Maywood, IL, USA; ^4^ Department of Public Health, Medical University of South Carolina, Charleston, SC, USA; ^5^ Department of Opthamology, Medical University of South Carolina, Charleston, SC, USA

**Keywords:** T cell, apoptosis, melanoma, immunotherapy, Immunology and Microbiology Section, Immune response, Immunity

## Abstract

Advancements in adoptive cell transfer therapy (ACT) has led to the use of T cells engineered with tumor specific T cell receptors, which after rapid expansion can be obtained in sufficient numbers for treating patients. However, due to massive proliferation these cells are close to replicative senescence, exhibit exhausted phenotype, and also display increased susceptibility to activation induced cell death. We have previously shown that tumor reactive T cells undergo caspase-independent cell death upon TCR restimulation with cognate antigen, which involves reactive oxygen species and c-jun N-terminal kinase. Herein, we show that a large fraction of the human melanoma epitope tyrosinase reactive TCR transduced T cells that exhibit effector memory (T_EM_) phenotype and undergo programmed necrosis, or necroptosis, upon TCR restimulation. As compared to the T central memory (T_CM_) subsets, the T_EM_ subset displayed an increased expression of genes involved in necroptotic cell death, and a necrotic phenotype upon TCR restimulation as confirmed by electron microscopy. Higher expression of receptor-interacting kinases (RIPK) that mediate necroptosis was also observed in the T_EM_ fraction. Further, the T_EM_ cells were rescued from undergoing necroptosis when pretreated with necroptotic inhibitor NecroX2 before TCR restimulation. Importantly, NecroX2 pretreated tumor reactive T cells also exhibited better tumor control and increased *in vivo* persistence when adoptively-transferred to treat subcutaneously established murine melanoma B16-F10. Thus, it is likely that the outcome of ACT could be vastly improved by interfering with the necroptotic cell death pathway in activated tumor reactive T cells used in immunotherapy.

## INTRODUCTION

Recent advancements in adoptive cell therapy (ACT) have led to the use of T cells engineered with tumor specific T cell receptors for targeting tumor growth [1]. This therapy requires about 10^9^ autologous T cells engineered with tumor reactive TCR that are injected to patients for obtaining a meaningful response. However, the rapid expansion protocol (REP) that is used to expand these TCR engineered T cells in the presence of anti-CD3 antibody and high concentration of cytokines often impart cells with an exhausted effector phenotype and increased susceptibility to cell death [1]. Although, which pathway of death do the tumor reactive T cell undergo is not well established, and could be important since there exists a tight correlation between the tumor control by ACT and ‘persistence’ [2]. Therefore, the focus of this study was to determine the pathway and signaling molecules involved in the death of tumor epitope reactive T cells used in ACT, and determine if blocking those pathways will result in improved tumor control.

Cell death can be executed by at least two well-established mechanisms, necrosis and apoptosis [3]. Recent studies have shown that necrosis may occur in a “programmed” manner just like apoptosis, and this form of necrotic death is now known as “necroptosis” [4, 5]. This form of cell death shares characteristics of both necrosis and apoptosis. While on one hand, necroptotic cells are morphologically similar to necrotic cells in that they undergo nuclear condensation and organelle swelling [6], on the other hand, necroptosis is a type of regulated cell death similar to apoptosis. It has been established that these cell death processes are interconnected and share regulatory mechanisms [3]. Underlying molecular mechanisms associated with necroptosis is dependent on the kinase activity of receptor-interacting protein kinase 1 (RIPK1) and receptor-interacting protein kinase 3 (RIPK3) [6–8]. It has been recently demonstrated that T cells may undergo necroptosis in the absence of caspase 8 and cell death may be orchestrated by RIPK1 and RIPK3 activity [9]. Importantly, Degterev *et al.,* (2005) also discovered that necroptotic cell death could be inhibited by a small molecule called necrostatin-1 (Nec-1). Nec-1 has been shown to be specific for necroptosis, but not apoptosis [5]. Thus, the multiplicity of cell death pathways and relevant proteins provides additional opportunities to develop new strategies for therapeutic inhibition of cell death.

Recent studies have also shown that non-caspase dependent death with necroptosis also involves c-jun N terminal kinase (JNK) and reactive oxygen species (ROS) pathways [10, 11]. Further, autophagic degradation of catalase resulting in increased accumulation of ROS that leads to JNK activation and eventually necroptotic cell death has also been shown [12, 13]. As mentioned above, we have earlier shown that epitope specific CTL underwent caspase independent activation induced cell death (AICD) upon TCR restimulation that was inhibited by employing JNK and ROS inhibitors [12–16]. Our data shows that majority of the tumor epitope reactive T cells exhibit necroptotic phenotype after repeated TCR stimulation, and using necroptosis inhibitor-pretreated T cells during adoptive T cell transfer therapy for melanoma results in increased T cell persistence that correlates with a robust tumor control. We believe that identifying the exact form of cell death that ensues on TCR restimulation in melanoma-specific CTL would help us identify better targets for intervention and cytoprotection that can translate into improved T cell immunotherapeutic regimens.

## RESULTS

### CD62L^lo^ T cells undergo necroptosis on TCR re-stimulation

**Figure 1:**
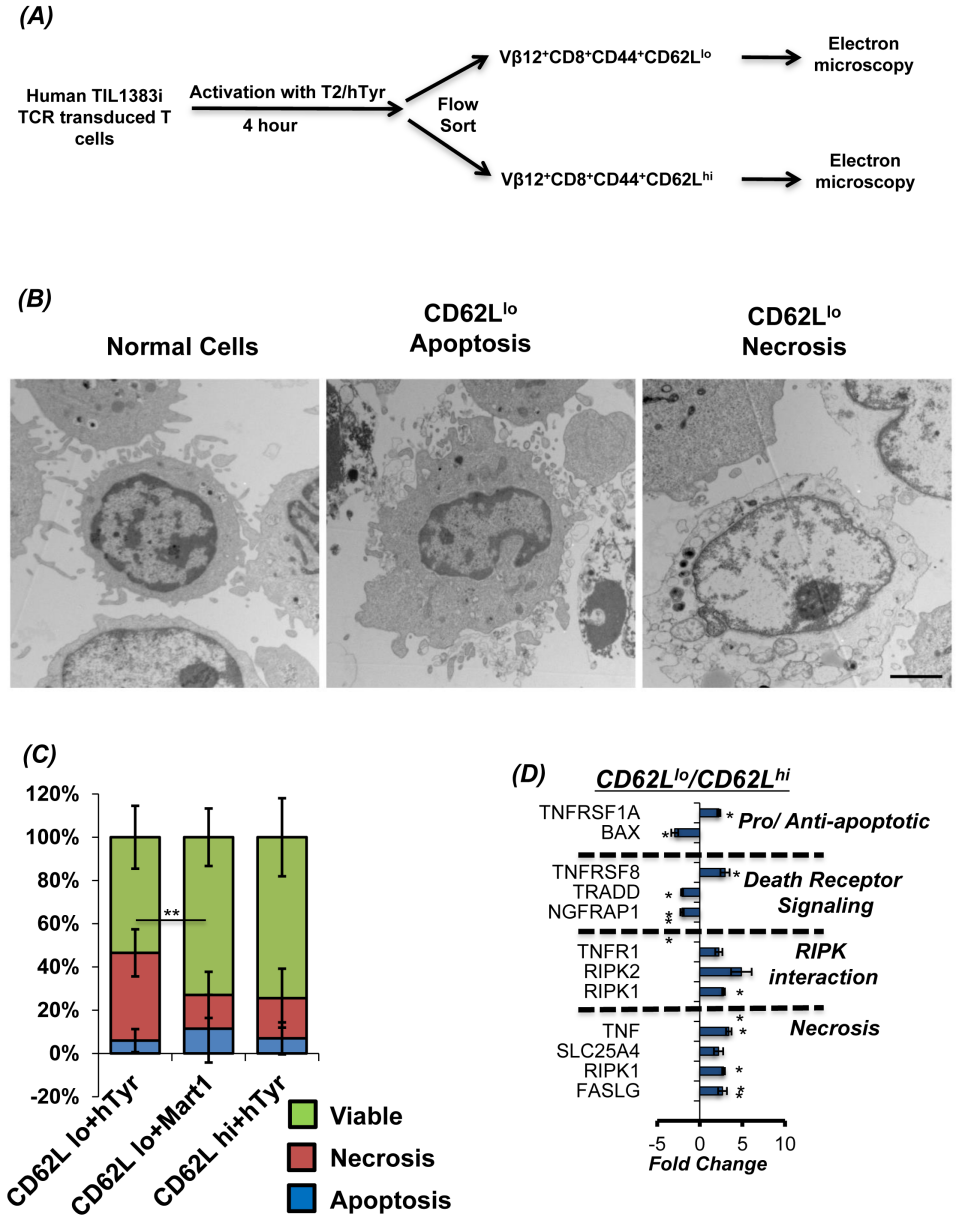
CD62L^lo^ cells show higher necrosis during AICD **A.** Schema for stimulation and sorting of these cells. Human TIL1383I TCR transduced cells were flow sorted after re-stimulating it with cognate peptide hTyrosinase (hTyr peptide presented on T2 cells - hTyr/T2) for 4 hours. The activated antigen reactive T cells were then sorted using FACS for CD8^+^CD44^hi^CD62L^hi^ and CD8^+^CD44^hi^CD62L^lo^ expression. These cells were further analyzed using electron microscopy (EM) and mRNA expression analysis. **B.** Electron microscopy (EM) pictures (at 8000X resolution) of T subsets undergoing apoptosis or necroptosis. Bar represents 2 micrometer. **C.** Eight pictures (3000x) from each sample were analyzed for necrosis, apoptosis or viable cells. Cumulative data is presented as bar diagram displaying the relative percentage of each phenotype (color coded) within a treatment group. The data shows results from two different samples and analysis. Red line shows the difference between necrosis between CD8^+^CD44^hi^CD62L^lo^ stimulated with right (hTyr) and wrong (Mart1) peptide. **D.** RNA from FACS sorted populations CD8^+^CD44^hi^CD62L^hi^ and CD8^+^CD44^hi^CD62L^lo^ cells were used for analysis of 84 different markers of necrosis and cell death using real-time PCR array. Results show data of 2 separate experiment. ** *p* <0.005; * *p* <0.05

Studies from our lab and others have shown that T cells with CD62L^lo^ phenotype do not persist longer *in vivo*, as compared to CD62L^hi^ T cells subsets [17, 18]. This difference in persistence is also directly correlated to the inability of CD62L^lo^ T cell subset to control tumor growth. While increased susceptibility to TCR re-stimulation induced cell death or oxidative stress induced cell death has been shown to be primarily responsible for decreased CD62L^lo^ T cell subset persistence [19], the kind of cell death that CD62L^lo^ T cell subsets undergo has not been comprehensively elucidated. While our previous data shows that tumor epitope reactive T cells undergo caspase-independent cell death that involves ROS and JNK [14, 15], we further evaluated the morphological changes associated with TCR restimulation cell death in CD62L^lo^ T cells (Figure 1A). Our data obtained using electron micrographs show that restimulation of the TIL1383I tyrosinase TCR transduced T cells with cognate antigen results in both necroptotic (Figure 1B, *extreme right panel*) and apoptotic phenotype in CD62L^lo^ (Figure 1B, *middle panel*). However, a quantitative enumeration showed that a major fraction of the T cells undergoing TCR restimulation cell death in CD62L^lo^ fraction exhibit necrotic phenotype (*red section of the bar in* Figure 1C), as compared to the apoptotic phenotype (*blue section of the bar in* Figure 1C). The CD62L^lo^ fraction that was stimulated with control peptide, exhibited less cell death indicating that induction of necroptosis is due to antigen specific TCR restimulation. Importantly, the CD62L^hi^ fraction that was restimulated with cognate peptide also displayed less cell death. Notably, this cell death was specifically due to antigen specific TCR restimulation (signal 1) as the surrogate antigen presenting cell T2-A2 that were used for restimulation also express co-stimulatory molecules (signal 2) (*supplementary figure 1*), and the co-culture was set-up in presence of IL2 cytokine (signal 3). Further, to confirm the differences in the morphology of cell death between the CD62L^lo^ *vs.* CD62L^hi^ fractions, the cognate antigen activated TCR transduced human T cells that were FACS sorted based on CD62L expression and mRNA was used for determining the death pathways with real-time PCR array (Qiagen Cell Death Pathway Finder PCR array, catalog # PAHS-212ZD). Our data in Figure 1D that shows gene expression analysis in CD62L^lo^/CD62L^hi^ fraction and confirms that TCR restimulation of the CD62L^lo^ T results in higher levels of RIP kinases and genes involved in a necrotic form of cell death. While we observed that the pro-apoptotic protein Bax involved in caspase mediated cell death was down regulated in the CD62L^lo^ cells, the expression of the tumor necrosis factor (TNF), and TNF receptor superfamily members such as TNFRSF1A, TNFRSF8, and TNFR1, was upregulated. The TNFR not only interact with TNF-α for NF-κB activation, but also interacts with RIPK1, TRAF, FADD and other proteins known to regulate necroptosis [6, 20]. Importantly, the upregulation of SLC25A4 (Solute Carrier Family 25 Adenine Nucleotide Translocator Member 4), which is also known as ADP/ATP translocator and exports ATP from the mitochondrial matrix and imports ADP was also found to be elevated. The change in ADP/ATP ratio is also implicated in different modes of cell death [21–23]. While increased levels of ATP and decreased levels of ADP identify proliferating cells, decreased levels of ATP and increased levels of ADP are recognized in apoptotic cells. Thus, an increased expression of SLC25A4 in CD62L^lo^ cells would imply a more profound decrease in ATP and increase in ADP - a hallmark of necrosis than apoptosis.

### Inhibition of necroptosis rescues TCR transduced human T cells from cell death

**Figure 2:**
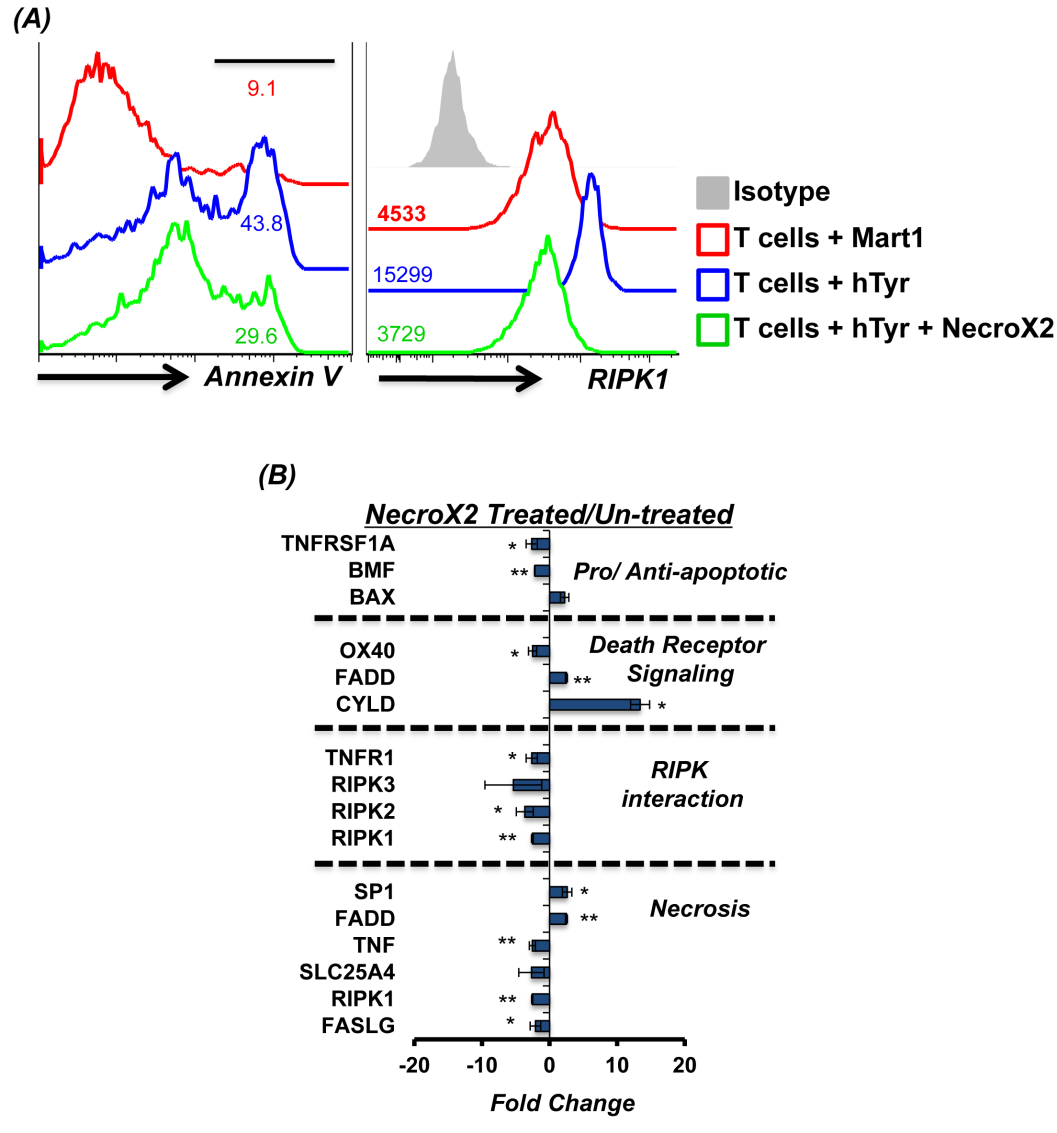
RIPK1 inhibitor can rescue T cells from AICD Human TIL1383I TCR transduced cells were re-stimulated using human tyrosinase peptide pulsed T2 cells or with control peptide Mart1 peptide pulsed T2 cells. In another group tumor reactive T cells were also pre-incubated for 40 minutes in RIPK1 inhibitor (1 μM NecroX2) and then re-stimulated with cognate peptide (hTyr) pulsed T2 cells. **A.** After 4 hr. of TCR restimulation, the T cells were analyzed for Annexin V upregulation and levels of RIPK1 using FACS **B.** After 4 hr. of TCR restimulation, the T cells were FACS sorted for CD8^+^ T cells and analyzed for 84 different markers of necrosis and cell death using real-time PCR array. A comparison was done between NecroX2 treated and untreated cells. The data shows cumulative results from two different samples and analysis. All results are representative of three or more separate experiments. ** *p* <0.005; * *p* <0.05

Given our data that majority of the human T cells engineered with tumor reactive TCR exhibit necroptotic phenotype upon restimulation with the cognate antigen, we tested if AICD could be rescued by blocking necroptosis. Thus, we used NecroX2, a cell-permeable necrosis inhibitor that localizes mostly to the mitochondria and selectively blocks oxidative stress-induced necrotic cell death [24]. Our data shows that human T cells pretreated with NecroX2 for 45 minutes before TCR restimulation exhibited reduced susceptibility to cell death as measured by Annexin V levels (Figure 2A, *left panel*). To confirm the necroptotic phenotype of the cells undergoing cell death [25], a parallel evaluation of RIPK1 was done. We observed that while TCR restimulation with cognate antigen (hTyr) increased RIPK1 expression, a decrease in RIP1K was noted when the activated T cells were pretreated with Necro-X2 before TCR restimulation (Figure 2A, *right panel*). NecroX2 pretreated TCR transduced T cells also showed reduced expression of genes involved in the necroptotic pathway as compared to those that were left untreated before TCR stimulation with cognate antigen (Figure 2B). This data confirms that the pharmacological inhibitor of necroptosis indeed inhibits the death pathway gene expression, and rescues human TCR transduced T cells from undergoing necroptotic cell death upon TCR re-engagement.

### NecroX2 pretreatment inhibits RIPK1-RIPK3 levels in TCR transduced CD62L^lo^ T cells

**Figure 3:**
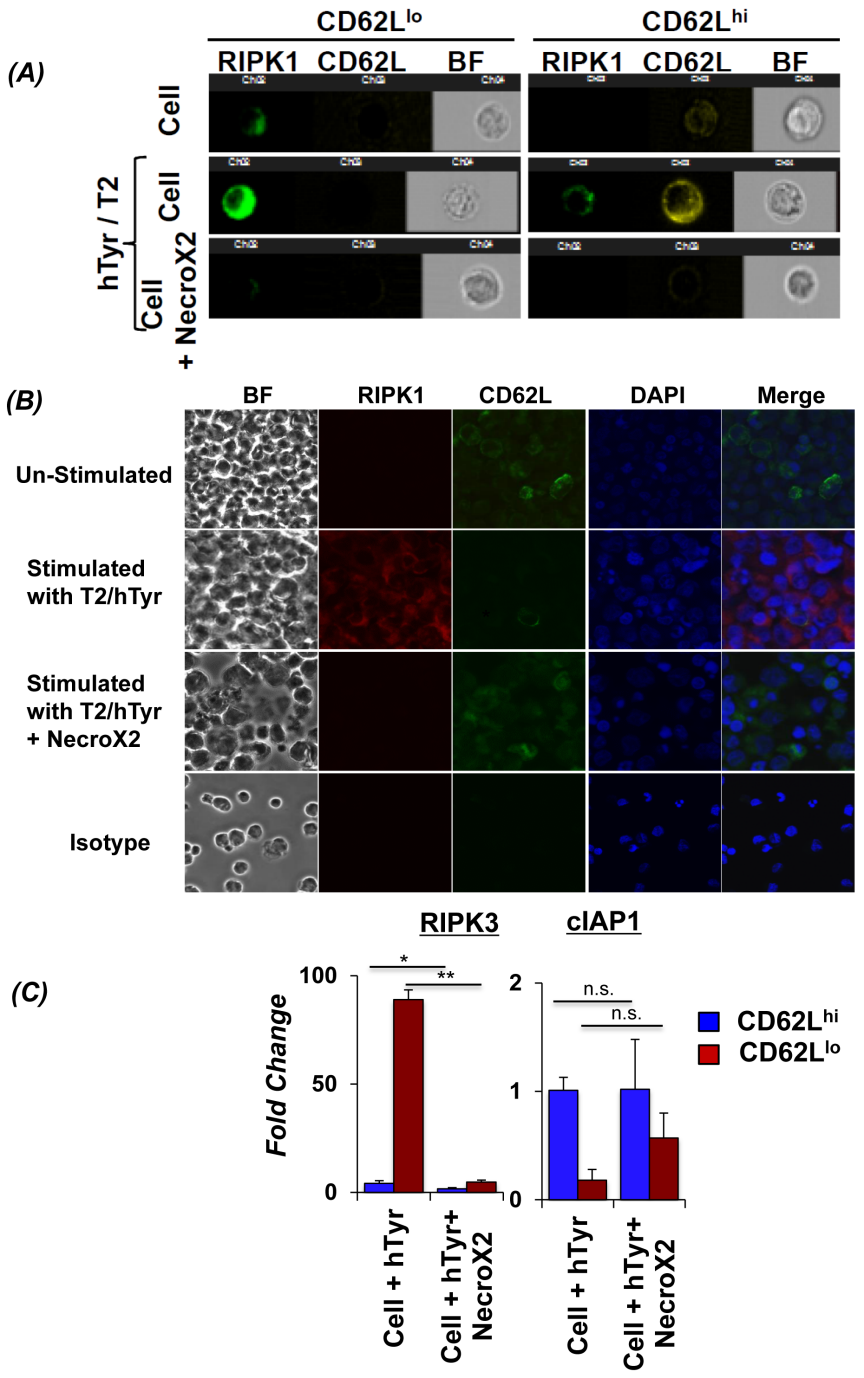
Inhibiting RIPK1 can rescue CD62L^lo^ CD8^+^ T cells from AICD Human TIL1383I TCR transduced cells were re-stimulated using cognate hTyrosinase (hTyr/T2) peptide or with control peptide (Mart1/T2). Cells were also pre-incubated in RIPK1 inhibitor (NecroX2 1μM) for 40 minutes and then re-stimulated using hTyr/T2. **A.** The cells from different treatment groups were analyzed for CD62L expression (Yellow), along with RIPK1 (Green) and bright field (BF) using Amnis Image Stream. Data was analyzed using IDEAS software (Amnis Corp). **B.** In another similar experiment that was set-up as describe above, CD8^+^T sorted 4 hr. after TCR restimulation were analyzed for expression of RIPK1 (Red) along with CD62L (Green) in the presence or absence of NecroX2 using confocal microscopy. Each experiment was repeated thrice and 10 different fields were analyzed in each experiment/slide. **C.** Human TIL1383I TCR transduced cells were re-stimulated using hTyrosinase peptide in presence or absence of NecroX2, and then FACS sorted CD8^+^CD44^hi^CD62L^hi^ and CD8^+^CD44^hi^CD62L^lo^ fractions were used for determining the mRNA expression of RIP3K and cIAP1. The pooled data presented is from three experiments with similar results. All results are representative of three to four separate experiments. ** *p* <0.005; * *p* <0.05.

In order to confirm for the role of RIP kinases specifically in CD62L^lo^ T cells, we used the Imaging based FACS platform (Amnis ImageStream) to gate the T cell subsets based on CD62L expression, and simultaneously evaluate the expression/localization of necroptosis mediating RIP kinases. Our data shows that TCR transduced human T cells gated for CD62L^lo^ expression exhibit higher levels of RIPK1 upon TCR restimulation (Figure 3A*, left panel*). However, T cells with CD62L^hi^ expression showed very low RIPK1 expression (Figure 3A*, right panel*). Importantly, NecroX2 pretreatment before TCR restimulation resulted in decreased RIPK1 expression in the CD62L^lo^ T cells (Figure 3A*, left panel, bottom row*). A cumulative analysis of thousand cells also showed significant differences in RIPK1 localization and CD62L expression. Similar observation for the RIP kinase co-expression with CD62L cells using microscopy was obtained when TCR transduced T cells were restimulated with (Figure 3B, second column second row) or without (Figure 3B, second column third row) NecroX2 pretreatment. Further, the RNA obtained from TCR activated T cells that were FACS sorted based on CD62L expression was used to determine the level of RIPK3 and cellular inhibitors of apoptosis (cIAP), other key proteins that are known to regulate necroptosis. Our data shows that CD62L^lo^ subsets showed many fold increased expression of RIPK3, and a concomitant decrease in cIAP1. However, when the cells were pretreated with NecroX2 before TCR restimulation, we observed that RIPK3 expression was decreased, whereas cIAP1 was upregulated (Figure 3C). Since cIAP1 is known to polyubiquitinate RIPK1 and leads to cell survival through downstream NF-κB signaling [26, 27], this data suggests that upon TCR restimulation human TCR transduced T cells undergo necroptotic cell death that involves upregulation of RIPK1, RIPK3, and inhibition of cIAP1.

### IFN-γ secretion directly co-relates to necroptosis

**Figure 4:**
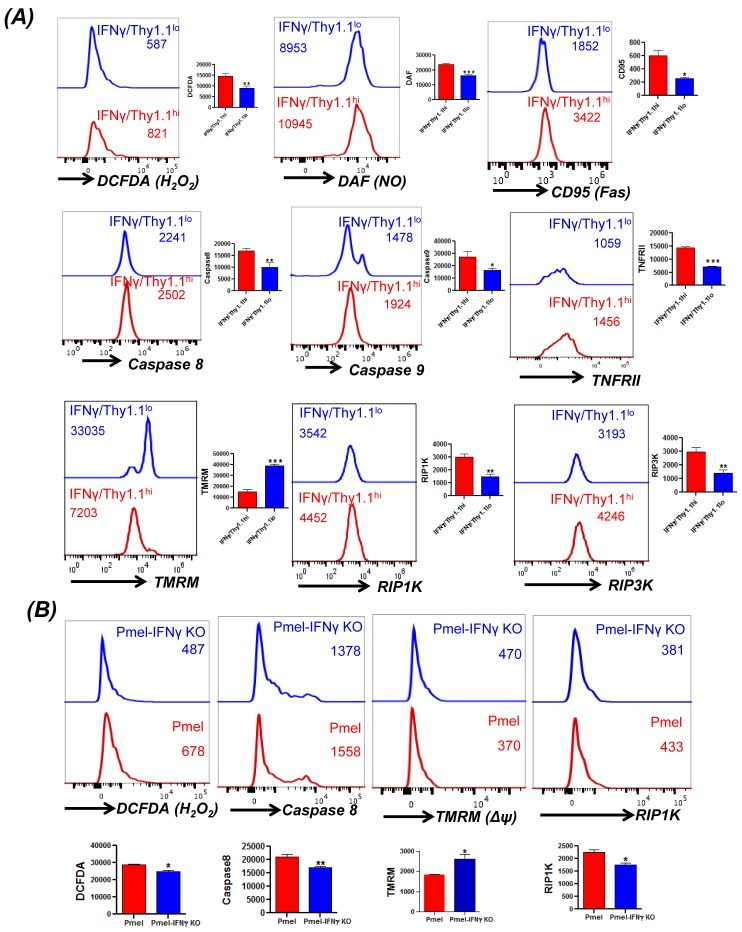
Direct correlation between IFNγ levels and cell death **A.** Splenic T cells from the IFNγ-Thy1.1 knock-in mice were TCR activated for three days using plate-bound anti-CD3/anti-CD28. Thereafter, the activated T cells were washed and TCR restimulated overnight using plate bound antibodies. FACS based analysis by staining for Thy1.1 expression that correlates to IFNγ levels was carried out for H_2_O_2_ (using DCFDA), NO (using DAF), CD95, caspase 8, caspase 9, TNFRII, mitochondrial membrane potential (using TMRM), and RIPK1, RIPK3 expression as detailed in methods. **B.** Splenic T cells from Pmel and Pmel-IFNγ KO mouse were activated for three days using cognate antigen gp100. Thereafter, the activated cells were restimulated with cognate gp100 antigen pulsed feeder cells for another 4 hr. Cells were stained and analyzed using FACS for H_2_O_2_ (using DCFDA), caspase 8, mitochondrial membrane potential (using TMRM), and RIP1K expression. Data presented is from one of three experiments with similar results.

Given our observation that T_EM_ phenotype bearing T cells that secrete higher level of effector cytokine IFNγ exhibit increased susceptibility to undergo necroptotic cell death, we next established if there is a direct link between IFNγ level and necroptosis. For this purpose we used the IFNγ-Thy1.1 knock-in mice (generously provided by Dr. Casey Weaver, UAB) [28]. The splenic T cells obtained from these mice allowed us to determine the fate of IFNγ^hi^ (≈Thy1.1^hi^) *versus* IFNγ^lo^ (≈Thy1.1^lo^) T cells. Our data in Figure 4A shows that TCR activated IFNγ^hi^ T cells exhibit increased ROS (as measured by DCFDA), increased RNS (as measured by DAF), increased loss of mitochondrial membrane potential (as measured by TMRM) upon TCR restimulation with anti-CD3/anti-CD28 antibody. IFNγ^hi^ T cells also exhibited increased levels of Fas (CD95), and TNFRII upon TCR restimulation. A recent study using the HIV epitope specific T cells has shown that increased cytoplasmic caspase-8 activity directly co-relates with the antigen exposure, whereas surface caspase-8 levels inversely correlat to TCR stimulation [29]. Importantly, we observed that the IFNγ^hi^ T cells that also exhibited increased cytoplasmic caspase-8 activity, (as measured by cell permeable fluorescently-labeled caspase-8 probe FAM-LETD-FMK that selectively binds only to active caspase-8 molecules), and higher levels of RIP1K/RIP3K. To confirm the above findings in a tumor antigen specific model, we also generated melanoma epitope gp100 TCR bearing transgenic mice Pmel on IFNγ-knockout background (Pmel-IFNγ KO). A comparison of spleen derived Pmel and Pmel-IFNγ KO T cells after antigen restimulation showed that Pmel-IFNγ KO T cells had reduced levels of ROS accumulation, caspase-8 activity and RIP1K activity (Figure 4B). Pmel-IFNγ KO T cells also showed reduced loss of mitochondrial membrane potential as measured using TMRM dye. This data suggests that T cells with the ability to secrete high level of effector cytokine IFNγ also secrete higher amount of ROS/RNS and have increased susceptibility for necroptotic cell death.

### Inhibiting necroptosis rescues tumor epitope reactive T cells

**Figure 5:**
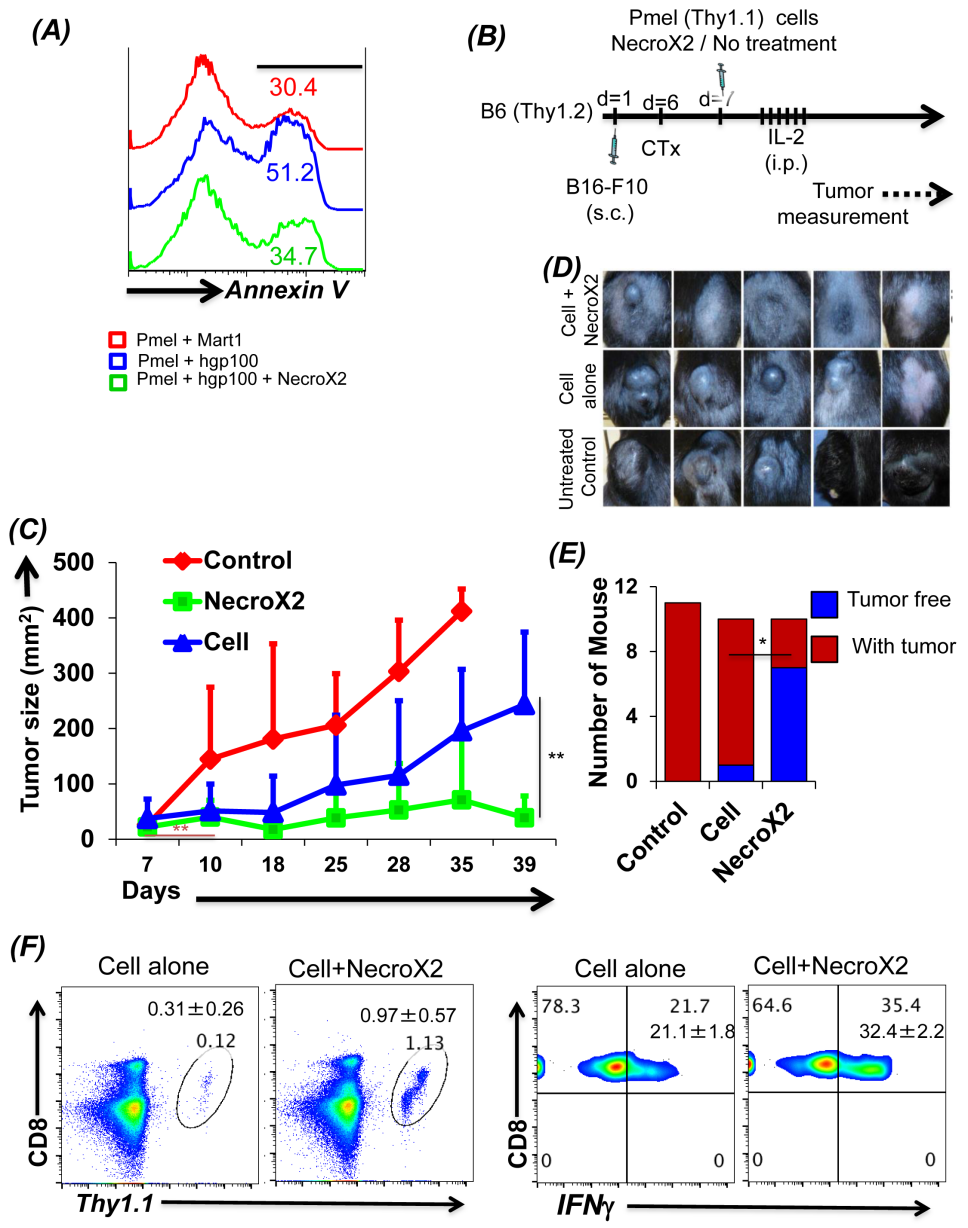
Inhibiting necroptotic death pathway rescues tumor reactive T cells from AICD and enhances tumor control **A.** CD8^+^ T cells from Pmel (Thy1.1) mouse were stimulated for 3 days using human gp100 peptide. Cells were then re-stimulated in antigen specific manner in presence or absence of NecroX2 (1 μM). Mart1/T2 was used a control peptide. T cells were analyzed for cell death using Annexin V staining. This experiment was replicated three to four times with similar results. **B.** Figure shows the schematic of the experiment. Pmel cells stimulated for 3 days were injected in B6 mice (Thy1.2) with B16-F10 tumors (7 day established tumors). Mouse was divided into three groups. One group was control and did not receive any adoptive transfer, second group received Pmel CD8^+^ T cells and the third group received Pmel CD8^+^ T cells along with dose of NecroX2 (*i.p.* from day 7 to 21 on alternate days) at the rate of 1.65 mg/kg/dose. **C.** Figure shows the tumor growth curves of mice detailed in *B*. **D.** Pictures show tumors of mice from one of the experiment on day 35. **E.** The graph represents number of mice having tumors (red) or with treated tumor (blue) at the end of the experiment (day 40). **F.** To analyze the presence of transferred cells, these mice were bled on day 35 and analyzed for Thy1.1^+^CD8^+^ cells. These cells were also analyzed for production of IFNγ cytokine after stimulating with PMA and Ionomycin. All results are representative of two separate experiments of 5-6 mice each. ** *p* <0.005; * *p* <0.05.

To further test the hypothesis that rescuing tumor epitope reactive T cells from undergoing necroptotic cell death will result in improved tumor control, we characterized the effect of pharmacological inhibitor NecroX2 on splenic T cells derived from Pmel mice. Our data in Figure 5A shows that gp100 reactive T cells undergoing necrotic cell death, when pretreated with NecroX2 before TCR restimulation exhibit reduced Annexin V upregulation. Notably, the expression of cell surface molecule CD62L and CD44 remain unchanged in presence of NecroX2 (*supplementary Figure 2a*), implying that it is only the intrinsic phenotype that is modulated in presence of this pharmacological inhibitor which rescues the CD62L^lo^ T cells from necroptotic death. We also observed that NecroX2 pretreatment resulted in reduced loss of mitochondrial membrane potential, reduced levels of JNK and RIPK1 (*data not shown*). This *ex vivo* characterization shows that inhibition of TCR restimulation mediated cell death could be rescued by blocking necroptotic cell death pathway. Next, for *in vivo* analysis the C57BL/6 mice with subcutaneously injected murine melanoma B16-F10 were treated by adoptively transferring gp100 TCR reactive T cells, which were either pre-treated with NecroX2 or not (Figure 5B). We observed that tumor bearing mice that were treated with NecroX2 pretreated T cells showed reduced tumor burden (Figure 5C, *green graph*), as compared to the ones that were treated with the T cell alone (Figure 5C, *blue graph;* and Figure 5D). Using NecroX2 alone did not control the tumor growth (*supplementary figure 2b*). The cumulative data from repeat experiments showed increased fraction of tumor free mice in the group that received T cells pre-treated with NecroX2 (Figure 5E). Tracking of transferred T cells from the tumor bearing recipient mice also confirmed that NecroX2 treatment affected the quantitative and qualitative phenotype of the T cells. While there was about ten-fold higher (*p* value ≤ 0.0018) number of cells in circulating peripheral blood at day 34 (Figure 5F, *left panel*), the NecroX2 treated T cells also secreted more (*p* value ≤ 0.0003) effector cytokine IFNγ (Figure 5F, *right panel).* Thus, our data firmly establishes that understanding the death pathway of effector T cells could be important in devising rationale strategies to target them for increased persistence and achieving better tumor control.

## DISCUSSION

The emerging complex interplay of apoptotic and non-apoptotic cell death mechanisms in animal models of human pathologies suggests that development of the approaches to inhibit apoptosis and, in many cases, non-apoptotic cell death, might be key to enhance the potential of cell based therapies. In this study, we examined AICD and confirmed that majority of the tumor epitope reactive T cells undergo necroptotic cell death upon TCR restimulation. We observed that the key molecules RIPK1 and RIPK3 are significantly upregulated during TCR restimulation, and blocking necroptotic cell death pathway using the pharmacological inhibitor rescues effector T cells from cell death, increases persistence *in vivo*, and lead to a vastly improved tumor control upon ACT. In addition to the TCR restimulation mediated necroptosis, this newly discovered pathway of regulated necrosis could be induced by numerous stimuli as death receptors, Interferons, Toll-like receptors, intracellular RNA and DNA sensors [30]. It has also been shown that human immunodeficiency virus type 1 (HIV-1) infection not only induces apoptosis, but also mediates necroptosis in the infected primary CD4^+^ T lymphocytes and CD4^+^ T-cell lines [31]. Treatment with necrostatin-1 (Nec-1), a RIPK1 inhibitor that specifically blocks the necroptosis pathway, potently restrains HIV-1-induced cytopathic effect. Another recent study has shown that chronic TCR stimulation contributes to dysfunctional HIV-specific CD8^+^ T cell proliferation through activation of necroptosis and increased cell death [29].

In addition to the morphological differences between a cell undergoing apoptosis *vs*. necroptosis, various mechanistic differences have been recently described as well [6]. An earlier study showed that necroptosis can be induced by CD95L, TRAIL or TNF in combination with Z-VAD-fmk, and is abrogated in RIPK1-deficient cells [32]. Later in 2005 Degretev *et al.* show that dimerization of RIP1 can induce necroptosis in FADD- deficient Jurkat T cells [5]. A recent study showed that T cell deficient in caspase 8 undergo necroptosis. It was also observed that T cells do not undergo necroptosis in RIPK3 deficient mouse [9]. Studies have shown that upon receiving death signal from TNFR and when caspase is inactive, RIPK3 binds to survival complex 1 (formed by RIPK1, cIAP, TNF receptor associated death domain (TRADD), TNF receptor-associated factor 2 (TRAF2) and TRAF5), acting as a switch to induce necroptosis [6, 33]. Our data shows that TCR mediated restimulation may preferentially target CD62L^lo^ T cells or CD8^+^ T_EM_ like cells with necroptotic cell death. We also observed that activation of RIPK1 is involved in necroptosis in CD62L^lo^ T cells. In addition, the T cells were rescued from AICD when RIPK1 inhibitors such as NecroX2 were used. We also observed that RIPK3 was also highly upregulated in CD62L^lo^ cells upon re-stimulation. Upon inhibiting the activity of RIPK1 we also observed a significant decrease in RIPK3 suggesting that cell death is being induced by RIPK1 and RIPK3 is also activated by TCR signaling. In addition, irrespective of the kind of stimulation (i.e. TCR based or using PMA/ionomycin) - the T cells underwent necroptotic cell death as indicated by increased expression of RIPK1/3 (*supplementary figure 3*). Admittedly, the expression of these kinases was less in PMA/ionomycin restimulated cultures as compared to the one that were TCR activated. This could be due to quantitative difference in phenotype of the T cells obtained with differences in primary stimuli (i.e. antigen versus PMA/ionomycin). Similar result showing that PMA/ionomycin can induce necroptosis has been detailed in another study [34], implying that the magnitude and morphology of cell death may depend more upon the of activation status and less upon the mode of restimulation. One study has also shown that AICD requires caspase 3, however AICD is independent of activator caspases such as caspase 8 and caspase 9 [35]. This further suggests that caspases 8 and 9 (major players of apoptosis) are inhibited during AICD and therefore cells may be using necroptosis machinery for undergoing AICD. One other important factor, which is responsible for inhibiting cell death, is cIAP. A recent study by Feoktistova *et. al*. reported that cIAP molecule inhibits ripoptosome (RIPK1, Fas associated death domain- FADD, caspase 8 complex) formation [36]. This suggests that cells when undergoing necroptosis should show lower levels of cIAPs. We observed in our study that cIAP1 was drastically reduced (>10 fold) in CD8^+^ CD62L^lo^ T cells. Therefore, we can infer that necroptosis may be important for AICD. This inference was further strengthened by the electron micrographs showing higher necroptosis in CD8^+^ CD62L^lo^ T cells.

One of the question that remains is as to what are the cumulative signals that determine the cell fate to die through apoptotic or necroptotic death pathway, and what is the physiological relevance of this form of death. A recent study has shown that RIPK1 and NF-κB signaling in dying cells determines cross priming of CD8^+^ T cells [37]. This implies that coordinated inflammatory and cell death signaling pathways within dying cells also contribute in orchestrating adaptive immunity, since decoupling NF-κB signaling from necroptosis or inflammatory apoptosis reduced priming efficiency and tumor immunity. Recent studies have also shown that the apoptotic and necroptotic death pathways could regulate each other [6]. Thus, it is likely that the difference between the TCR signaling thresholds in CD62L^lo^ *vs.* CD62L^hi^ T cells could determine the commitment to an apoptotic *vs.* necroptotic form of cell death. While the cytokine TNF and TNF family receptors have been implicated in necroptotic cell death [6], recent studies have also established the role of IFNγ in necroptosis [38]. This study showed that IFNγ induces RIP1K-dependent necroptosis in mammalian cells by inducing progressive accumulation of ROS and eventual loss of mitochondrial membrane potential in cells deficient in NF-κB signaling. They also identified the antioxidant enzyme manganese superoxide dismutase (MnSOD), which is encoded by SOD2 and quenches ROS, as a potential anti-necroptotic gene. Using IFNγ-Thy1.1 knock-in mouse derived T cells we observed that upon TCR activation the IFNγ^hi^ cells showed higher accumulation of ROS, RNS, and TNFRII. The loss of mitochondrial membrane potential was also more pronounced in IFNγ^hi^ cells as compared to the IFNγ^lo^ cells. This is in line with our previous observation where increased expression of reduced thiol groups and anti-oxidant capacity correlated with the low cytokine secreting CD62L^hi^ T cells [17]. In addition, the increased expression level of RIPK1, ROS, TNFRII or loss of membrane potential was reduced when the T cells obtained from the Pmel-IFNγ KO mouse were restimulated with the cognate antigen. These set of data implicate that tumor antigen reactive T cells with higher capacity to produce IFNγ have a direct co-relation to increased susceptibility to cell death, and a majority of these cells undergo necroptotic death upon repeated TCR activation. Recent studies that have shown that blocking glycolytic commitment by pretreating T cells with 2-deoxy glucose (2-DG) also renders the T cells with reduced IFNγ secreting ability, but improved *in vivo* survival and tumor control [39]. Similarly, T cells with lower mitochondrial membrane potential exhibit increased stem cell like phenotype [40]. Thus, it is likely that decreased persistence of the IFNγ^hi^ T cells is reverted by inhibiting the necroptotic cell death pathway, leading to improved tumor control. We believe that our observation that significant fraction of anti-tumor effector T cells could undergo “necroptosis” is translationally significant, and interfering with this dominant form of cell death could be of immediate value in adoptive cell therapy trials for increasing persistence and enhanced tumor control.

## MATERIALS AND METHODS

### Cells, culture medium, and reagents

PBMCs from healthy donors were obtained from a commercial vendor, Research Blood Components, LLC (Brighton, MA), after institutional approval by the Human Investigation Review Board. Culture medium was Iscove's Modified Dulbecco's Medium (GIBCO BRL, Grand Island, NY) supplemented with 10% fetal bovine serum (Gemini Bioproducts, Inc., Calabasas, CA). Ficoll-Paque was obtained from Amersham Biosciences (Piscataway, NJ). Recombinant interleukin (IL)-15 and IL-2 were purchased from R & D Systems (Minneapolis, MN). Fluorochrome-conjugated Annexin-V and monoclonal antibodies were obtained from BD Biosciences (San Jose, CA) or from BioLegend (San Diego, CA).

### Animals and cell lines

C57BL/6 and Pmel mice were purchased from Jackson Laboratory, and stocks were maintained at MUSC animal facility in pathogen-free facilities under the approved procedures of the Institutional Animal Care and Use Committee. T2-A2 cells are transporter-associated protein-deficient and its empty surface HLA-A2 molecules were used for direct presentation of epitopes to the antigen-reactive CTL. B16-F10 (H-2^b^) is a tyrosinase-positive murine melanoma.

### Confocal microscopy

Human TCR transduced cells (TIL1383I) were re-stimulated in the presence or absence of NecroX2. Cells were stained with human RIP1 kinase (Cell signaling technologies) and CD62L (BioLegend), following which cells were concentrated on the microscopic slides using a cytospin centrifuge. A coverslip was placed over the cells in mounting media containing DAPI. Ten different fields were imaged in both red and DAPI channels for each sample using the Olympus FV10i (Olympus, Center Valley, PA) confocal microscope equipped with a 60X water immersion objective n.a. 1.3. ImageJ software (National Institutes of Health) was used to analyze the images.

### Adoptive T cell transfer experiment

For adoptive transfer experiments, C57BL/6 mice were injected with B16-F10 tumors cells subcutaneously and allowed to establish for at least 7 days. Naïve splenocyte from Pmel mice were isolated and activated *ex vivo* using hgp100 peptide_25-33_ (KVPRNQDWL1 μg/mL) in presence of 50 IU/ml rhIL-2 for three days. For lymphodepletion, cyclophosphamide (4mg/mouse) was administered intraperitoneally (*i.p.*) 24hrs before adoptive transfer. Equal number of CD8^+^Vβ13^+^ cells (~1-2x10^6^) were transferred in each mouse. Interleukin-2 (rhIL-2 = 20 μg/dose) was given twice daily *i.p.* for 3 days. NecroX2 group received cells pretreated treated with 1 μM of NecroX2. NecroX2 treatment group mice also received necroX2 *i.p.* (1.65 mg/kg) for 2 weeks every alternate day.

### Engineering of TCR-transduced human T cells

Tyrosinase_368-376_ reactive TCR transduced T cells were generated as described earlier [41]. Briefly, using TCR was isolated from tumor infiltrating lymphocytes (TIL)TIL 1383I) [42] and was transfected into T cell using retroviral vector encoding (TIL1383I) tyrosinase epitope (YMDGTMSQV) specific TCR, and a truncated version of the human CD34 molecule (CD34t), as a selection marker.

### Image stream analysis

Human CD8^+^ TCR transduced cells (TIL1383I TCR) cells were stained with CD62L and RIPK1 antibodies (Cell signaling technologies). The experiment was repeated 2-3 times. Cells were acquired on Image stream (Amnis Corporation, Seattle, WA). Data from image stream was analyzed on IDEAS v5.0 (Amnis Corporation, Seattle, WA) by gating on either CD62L^hi^ or CD62L^lo^ T cells and then comparing RIPK1 intensity in them.

### Real-time PCR

A relative change in the expression of each gene compared to the control cultures from 3 different donors was considered to be significant. Real time for individual genes was done using Sso advance SYBR green (Bio-Rad Hercules, CA). Primers sequences for individual genes can be provided on request.

### Statistical analysis

All data reported are the arithmetic mean from three or five independent experiments performed in triplicate ±SD unless stated otherwise. Unpaired Student's *t*-test was used to evaluate the significance of differences observed between groups, accepting *p* < 0.05 as a threshold of significance. Data analyses were performed using the Prism software (GraphPad, San Diego, CA). *In vivo* data were analyzed using Kaplan-Meier methods and pairwise comparisons of survival distributions were done via the log-rank test. Mice that did not reach a tumor size of 400 mm^3^ by the end of the experiment were sacrificed and had survival time censored in the analysis.

The work was supported in part by NIH grants R21CA137725 and R01CA138930 to SM, and P01CA154778 to MIN.

## SUPPLEMENTARY MATERIAL


