# Supplementary material for: Blocking TCR restimulation induced necroptosis in adoptively transferred T cells improves tumor control
Source: Oncotarget. 2016 Oct 14;7(43):69371–83. doi: 10.18632/oncotarget.12674 (PMC5342484; doi:10.18632/oncotarget.12674)
Supplement: Supplementary file 1 [file oncotarget-07-69371-s001.pdf]

Blocking TCR restimulation induced necroptosis in adoptively transferred T cells improves tumor control

Supplementary Material

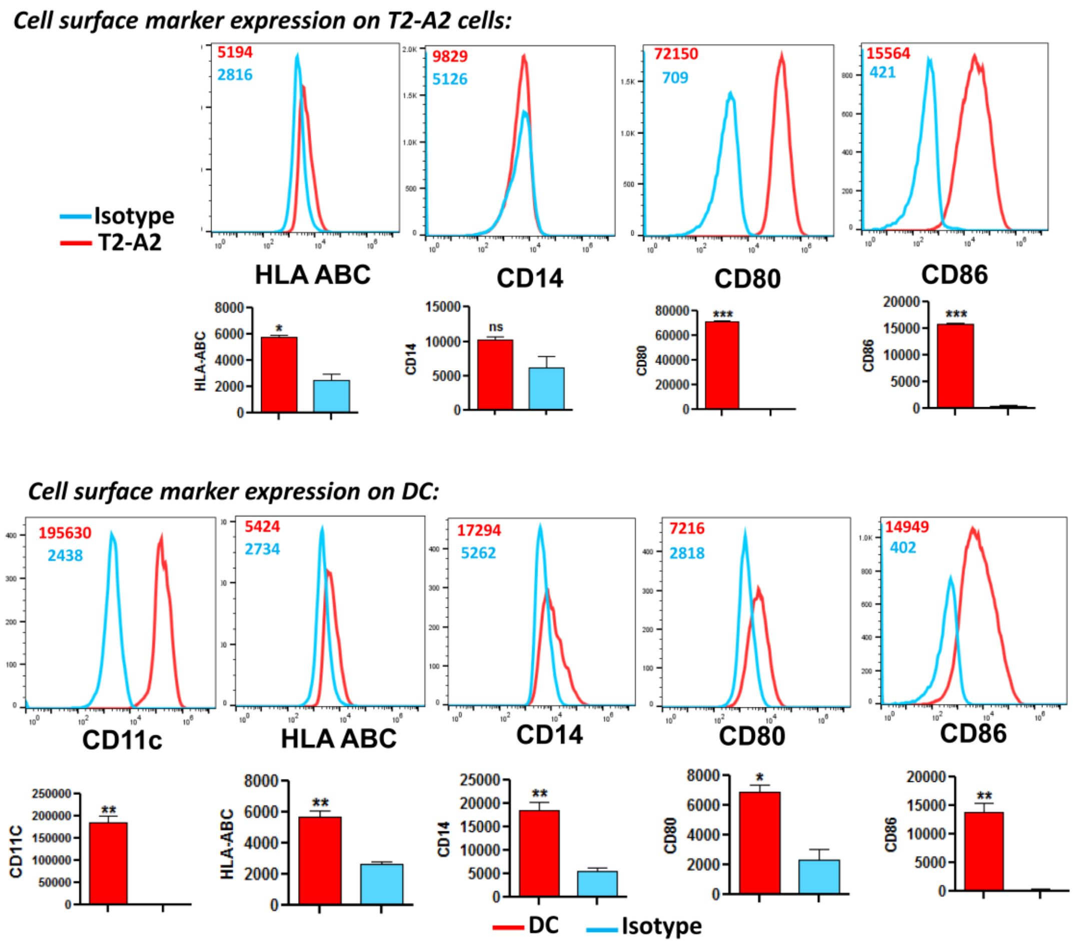

Supplementary Figure 1: **T2-A2 cells express co-stimulatory molecules**. Transporter associated protein deficient T2-A2 cells that were used as surrogate antigen presenting cells for stimulating the TIL1383I TCR transduced human T cells after direct loading of the peptide were used for cell surface expression of various co-stimulatory molecules using flurochrome conjugated antibodies. The *upper panel* shows expression of antigen presenting molecule HLA-ABC, monocyte marker CD14, and co-stimulatory molecules CD80 and CD86. A comparison phenotype was performed with the

professional antigen presenting immature dendritic cells (DCs) generated from human monocytes in GM-CSF and IL4 (*lower panel*). The numerical values on the upper left corner indicate the mean fluorescence intensity (MFI). The bar diagram below each molecule in both panels is the cumulative data from different experiments. *p value <0.05, ** p value < 0.01.

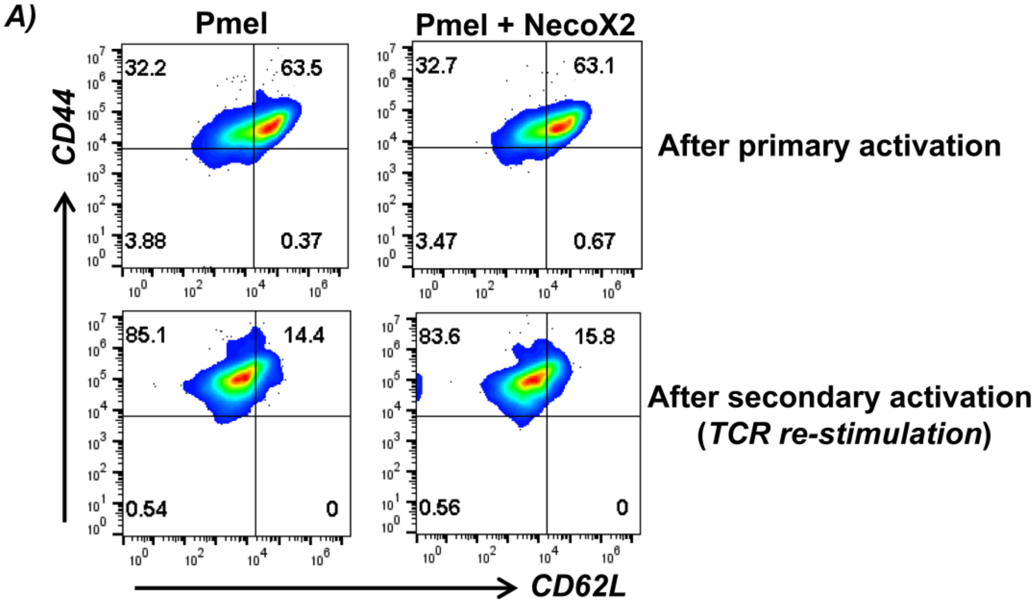

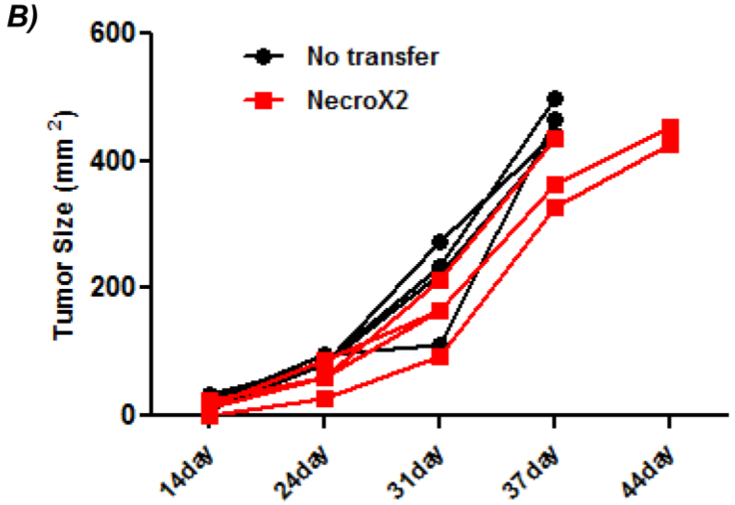

Supplementary Figure 2: **NecroX2 alone does not affect cell surface phenotype or tumor growth.** *A)*. $CD8^+$ T cells from Pmel transgenic mouse were stimulated for three days using gp100 peptide in presence or absence of NecroX2 (1 µM). Cells were then re-stimulated similarly with cognate antigen, and stained for different cell surface markers. Thereafter, gated gp100 specific $V\beta13^+$ $CD8^+$T cells were analyzed for CD62L and CD44 expression. *Upper panel* represents the phenotype of the three day activated T cells (or after primary activation), while the *lower panel* is the phenotype after the TCR restimulation (or secondary stimulation) of the activated T cells. *B)*. The C57BL/6 recipient mice with sub0cutaneously established murine melanoma B16 were either left untreated or treated with NecroX2 (*i.p.* from day 7 to 21 on alternate days) at rate of 1.65 mg/kg/dose. Tumor growth curve for three mice per group is shown.

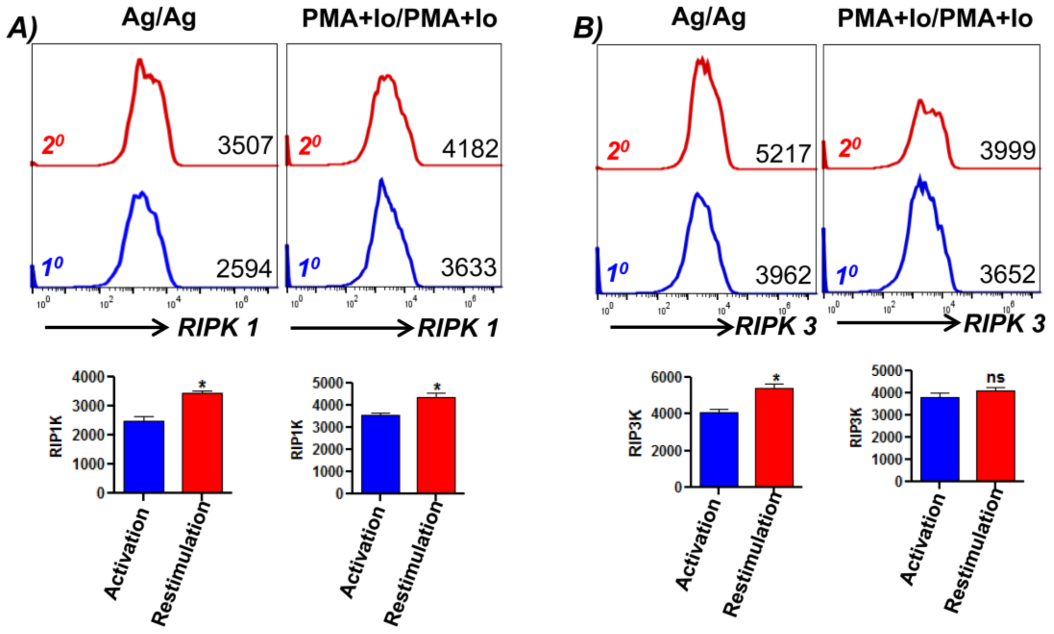

Supplementary Figure 3: **T cells undergo necroptosis upon restimulation.** $CD8^+$ T cells from Pmel transgenic mouse were stimulated for three days using either cognate antigen (Ag) gp100 or PMA + Ionomycin (10ng/ml). After primary stimulation (1^0), the cells were restimulated again (2^0 stimulation) with the same stimuli. Thus, Ag stimulated was restimulated with Ag (Ag/Ag), and PMA+Io stimulated were restimulated with PMA+Io (PMA+Io/PMA/Io). Cells were then stained for different markers and finally $V\beta13^+$ $CD8^+$ T cells were analyzed for RIPK1 and RIPK3 expression using FlowJo software. The overlapping histogram from 1^0 and 2^0 populations obtained after different stimulation are shown for RIPK1 expression *(A),* and RIPK3 expression *(B).* The numerical values adjacent to each histogram indicate the mean fluorescence intensity (MFI). The bar diagram below each molecule in both panels is the cumulative data from different experiments. **p* value <0.05.
